# Comparison of First-Pass Effect in Aspiration vs. Stent-Retriever for Acute Intracranial ICA Occlusion

**DOI:** 10.3389/fneur.2022.925159

**Published:** 2022-06-30

**Authors:** David Hernández, Elena Serrano, Gemma Molins, Federico Zarco, Oscar Chirife, Mariano Werner, Blanca Lara, Anna Ramos, Laura Llull, Manuel Requena, Marta de Dios las Cuevas, Sebastián Remollo, Carlos Piñana, Antonio López-Rueda

**Affiliations:** ^1^Hospital Universitario Valle de Hebrón, Barcelona, Spain; ^2^Hospital Clínic de Barcelona, Barcelona, Spain; ^3^Institute for Medical Engineering and Science (IMES), Massachusetts Institute of Technology (MIT), Cambridge, MA, United States; ^4^Hospital Universitario de Bellvitge, Hospitalet de Llobregat, Barcelona, Spain; ^5^Hospital Universitario Germans Trias i Pujol, Barcelona, Spain

**Keywords:** stent retriever, thrombectomy, internal carotid artery occlusion, revascularization, endovascular

## Abstract

The purpose of this study is to evaluate the best endovascular approach (aspiration or stent-retriever) and the impact of stent retriever size and length on clinical and angiographic outcomes in patients with acute intracranial ICA occlusion. We conducted a retrospective analysis of a prospective database of consecutive patients with acute intracranial ICA occlusion undergoing endovascular treatment in four Comprehensive Stroke Center between June-2019 and December-2020. We include 121 patients; Stent-retriever (SR) was used as first technical approach in 107 patients (88.4%) and aspiration was used in 14 patients (11.6%). SR group had higher rate of FPE compared to aspiration group (29 vs. 0%, *p* = 0.02). In SR subgroup, treatment highlighted higher FPE in the 6 × 50 SR (37.7%), than in the rest of the SR which are 21.2% (4–5 mm size and 20–50 mm length SR) and 19% (6 mm size and 25–40 mm length SR), but it was not found to be statistically significant. There were no other significant differences across the groups regarding primary angiographic or clinical outcomes. In our intracranial ICA occlusion series, stent retrievers were superior to direct aspiration in obtaining FPEs and mFPEs, and longer devices achieved better results with no statistically significant difference. Further studies evaluating the effects of different ICA clot removal approaches are warranted to confirm these results.

## Introduction

Acute ischemic stroke (AIS) is a complex disease encompassing multiple subtypes with different underlying pathologies. Acute occlusion of the internal carotid artery (ICA) is one of the most devastating events, with an incidence of 6–15% in patients with AIS (1). Patient outcomes are poor with only 2–12% achieving good recovery, 40–69% experiencing permanent severe neurological deficits, and 16–55% dying from AIS (2). Recanalization rates after intravenous (IV) and intra-arterial (IA) therapy ranged from 4.4 to 12.5% and from 62 to 63%, respectively. The high clot burden and limited delivery of recombinant tissue plasminogen activator (rt-PA) to intracranial occlusions may explain the poor prognosis (3, 4).

Complete revascularization from a single thrombectomy device pass, known as the first-pass effect (FPE), is associated with higher rates of good clinical outcomes. Usually, it is less likely to be achieved in internal carotid artery terminal occlusion (5). There is no consensus on the most effective endovascular technique for the treatment of acute intracranial ICA occlusion, aspiration did not demonstrate higher recanalization rates compared to stent-retriever in the anterior circulation occlusions (6–9). This study aimed to evaluate the best endovascular approach (aspiration or stent retriever) and the impact of stent retriever size and length on clinical and angiographic outcomes in patients with acute intracranial ICA occlusion.

## Materials and Methods

### Study Design and Patient Population

We conducted a retrospective analysis of a prospective database of consecutive patients with acute intracranial ICA occlusion undergoing endovascular treatment at four comprehensive stroke centers between June 2019 and December 2020. All participating centers received institutional review board approval from their respective institutions and patients or representatives and signed informed consent for the endovascular procedure and data analysis.

The inclusion criteria were patients aged ≥18 years, acute intracranial ICA occlusion confirmed on digital subtraction angiography, time from last seen well to treatment up to 24 h, baseline National Institutes of Health Stroke Scale (NIHSS) score ≥2, and premorbid modified Rankin Scale (mRS) score ≤ 3. Exclusion criteria include patients without available data to demonstrate efficacy or safety variables. No other exclusion criteria were addressed.

### Demographic, Clinical, and Radiological Data

The patient demographics included age and sex. Clinical and radiological data included previous modified Rankin scale (mRS), National Institutes of Health Stroke Scale (NIHSS) score, administration of intravenous thrombolysis with rt-PA, presence of tandem extracranial lesions, laterality, and evidence of early ischemic changes on non-enhanced computed tomography (NECT) with ASPECTS (Alberta Stroke Program Early CT Score).

### Procedural Data

Procedural data included the time from last seen well to the groin puncture, first intracranial series, and final revascularization series. The type of anesthesia (local, sedation, or general), ICA recanalization after the first pass, number of passes to carotid recanalization, and final ICA recanalization were analyzed. Intracranially, the modified thrombolysis in cerebral infarction (mTICI) score after the first pass, the final number of passes, and the mTICI final score were collected. The primary angiographic outcomes were the rate of ICA recanalization after the first pass, first-pass effect (FPE) (defined as mTICI ≥2c, achieved after the first attempt), and modified first-pass effect (mFPE) (defined as mTICI ≥2b, achieved after the first attempt). The mTICI score, FPE and mFPE results are related to MCA territory.

Procedure-related complications such as distal embolism, arterial perforation (defined as angiographic contrast extravasation that occurred during the procedure), or arterial dissection (described as the presence of an intimal flap on the control angiogram obtained after thrombectomy) were also documented.

Regarding the technical approach, angioplasty and/or carotid stent placement in tandem occlusions as well as antiplatelet management, the use of balloon guide catheter (BGC), intermediate catheter, and stent retriever (size and length) were up to the treating neurointerventionalist' choice and were recorded, as well as the change to other material during the procedure. The use of stentriever with or without intermediate catheter were also up to the treating neurointerventionalist' choice. The aspiration technique was performed with large bore catheters and manual aspiration in the vast majority of patients.

### Clinical Outcome Data

The primary clinical outcome was the mRS score at 90 days post-procedure, and a favorable outcome was defined as an mRS score from 0 to 2. Safety evaluations included assessment of the NIHSS score at 24 h and the presence of symptomatic intracranial hemorrhage (defined as a documented hemorrhage associated with a decline of four or more points in the NIHSS score).

### Statistical Analysis

Descriptive analyses included frequencies and percentages for categorical variables and means [standard deviations (SDs)] or medians [interquartile ranges (IQRs)] for continuous variables. Endovascular approaches were dichotomized into aspiration or stent-retriever thrombectomy, and trichotomized in the stent-retriever group according to the size and length of the device. Primary angiographic and clinical outcomes were compared between different endovascular approaches. Student's *t*-test or Wilcoxon's rank-sum test for continuous variables and the chi-squared (χ^2^) test or Fisher's exact test for categorical variables were performed using the Statistical Package for the Social Sciences (SPSS) software for Windows version 20.0 (IBM SPSS, SPSS Inc., Chicago, Illinois, USA). Statistical significance was set at *p* <0.05.

## Results

We included 121 patients from four comprehensive stroke centers between June 2019 and December 2020. We did not exclude any patients because of the lack of available data. The demographic, clinical, radiological, procedural, and clinical outcome data are summarized in Table 1. Regarding procedural safety variables, 23 patients (19%) experienced complications during the procedure. Sixteen patients (13.2%) had a distal embolism to the anterior or middle cerebral artery, five patients (4.1%) experienced arterial perforation during the procedure, and two patients (1.7%) presented with dissection of the internal carotid artery during the endovascular treatment.

**Table 1:** Demographic, clinical, radiological, and procedural data.

	**Patients (*n* = 121)**	**Stent-retriever (SR) (*n* = 107)**	**Direct aspiration (DA) (*n* = 14)**	***p*** **-value**
Age [years], median (IQR)	78 (70–86)	78 (70–86)	75 (70–82)	0.345
Female gender, *n* (%)	72 (59.5)	62 (57.9)	10 (71.4)	0.397
Previous mRS score, *n* (%)				0.112
0	62 (51.2)	52 (48.6)	10 (71.4)	
1	29 (24)	28 (26.2)	1 (7.1)	
2	21 (17.4)	18 (16.8)	3 (21.4)	
3	9 (7.4)	9 (8.4)	0 (0)	
NIHSS score at admission, median (IQR)	19 (16-22)	19 (16-22)	18 (14-22)	0.442
ASPECTS at admission, median (IQR)	8 (7-10)	8 (7-10)	9 (7-10)	0.405
Unknown time of symptom onset, *n* (%)	54 (44.6)	46 (43)	8 (57.1)	0.395
Last time seen well (min), median (IQR)	201.5 (124.5–330)	164 (95–262)	237 (116–332)	0.275
IV tPA administered, *n* (%)				0.810
None	82 (67.8)	73 (68.2)	9 (64.3)	
Partial dose	12 (9.9)	11 (10.3)	1 (7.1)	
Completed dose	27 (22.3)	23 (21.5)	4 (28.6)	
Type of anesthesia				0.308
General anesthesia, *n* (%)	23 (19)	19 (17.8)	4 (28.6)	
Local anesthesia, *n* (%)	24 (19.8)	23 (21.5)	1 (7.1)	
Conscious sedation, *n* (%)	74 (61.2)	65 (60.7)	9 (64.3)	
Left site occlusion, *n* (%)	64 (52.9)	58 (54.2)	6 (42.9)	0.571
Tandem occlusion, *n* (%)	14 (11.6)	13 (12.1)	1 (7.1)	1
Groin puncture to first run [min], median (IQR)	10 (5-15)	10 (5-13)	11 (5-26)	0.111
Groin puncture to revascularization [min], median (IQR)	48 (30–75)	49 (28–70)	35 (28–120)	0.942
Final number of passes to ICA recanalization, *n* (%)				0.345
1	67 (55.4)	60 (56.1)	7 (50)	
2	30 (24.8)	27 (25.2)	3 (21.4)	
>2	17 (18.2)	16 (14.9)	1 (7.1)	
ICA recanalization, *n* (%)	114 (94.2)	103 (96.3)	11 (78.6)	0.033
Final number of passes, *n* (%)				0.204
1	33 (27.3)	32 (29.9)	1 (7.1)	
2	35 (28.9)	28 (26.2)	7 (50)	
>2	53 (43.8)	47 (43.9)	6 (42.9)	
Final mTICI score, *n* (%)				0.432
0	9 (4.6)	6 (5.6)	3 (21.4)	
1	2 (1.7)	2 (1.9)	0 (0)	
2a	5 (1.4)	5 (4.7)	0 (0)	
2b	18 (14.9)	16 (15)	2 (14.3)	
2c	26 (21.5)	23 (21.5)	3 (21.4)	
3	61 (50.4)	55 (51.4)	6 (42.9)	
Symptomatic intracranial hemorrhage, *n* (%)	15 (12.4)	14 (13.1)	1 (7.1)	0.697
Complications, *n* (%)	23 (19)	22 (20.6)	1 (7.1)	0.303
NIHSS score at 24 h, median (IQR)	14 (5-21)	14 (5-21)	1414 (5-22)	0.951
mRS score at 3 months, *n* (%)				0.958
0–2	38 (31.4)	34 (31.9)	4 (28.6)	
3–5	47 (38.8)	30 (37.3)	7 (50)	
6	36 (29.8)	33 (30.8)	3 (21.4)	

Regarding the technical approach, a BGC was used in 45 patients (37.2%), an intermediate catheter in 108 patients (89.3%), and a stent retriever in 107 patients (88.4%). Direct aspiration (DA) was used as the first technical approach in 14 patients (11.6%) and a stent retriever (SR) was used in the remaining patients. Out of the 107 remaining patients in whom the stent retriever was used (88.4%), in 53 patients (43.8%), a 6 × 50 stent retriever was the one of choice; in 21 patients (17.4%) a 6 mm diameter and 25–40 mm long stent-retriever size was used; in 33 patients (27.3%) a 4–5 mm stent-retriever size was used.

The primary endovascular and clinical outcomes related to the endovascular approach are summarized in Tables 2, 3, respectively. Stent-retriever group had higher rate of FPE compared to aspiration group (29 vs. 0%, *p* = 0.02). There was no statistically significant difference regarding demographic, radiological and clinical data between stentretriever and direct aspiration groups.

**Table 2:** Primary endovascular and clinical outcomes related to the endovascular approach.

	**Overall (*n* = 121)**	**Stent-retriever (SR) (*n* = 107)**	**Direct Aspiration (DA) (*n* = 14)**	***p*** **-value**
ICA recanalization after first pass, *n* (%)	69 (57)	62 (57.9)	7 (50)	0.581
FPE (TICI 2c-3), *n* (%)	31 (25.6)	31 (29)	0 (0)	0.020
mFPE (TICI 2b-3), *n* (%)	40 (33.1)	38 (35.5)	2 (14.3)	0.136
Favorable Outcome (0–2 mRS score at 3 months), *n* (%)	38 (31.4)	34 (31.8)	4 (28.6)	1

**Table 3:** Primary endovascular and clinical outcomes related to the stent-retriever size and length.

	**Overall (*n* = 10,721)**	**6 mm size 50 mm length (*n* = 53)**	**4-5 mm size 20–50 mm length (*n* = 33)**	**6 mm size 25–40 mm length (*n* = 21)**	***p*** **-value**
ICA recanalization after first pass, *n* (%)	62 (57.9)	33 (62.3)	18 (54.5)	11 (52.4)	0.448
FPE (TICI 2c-3), *n* (%)	31 (29)	20 (37.7)	9 (21.2)	4 (19)	0.081
mFPE (TICI 2b-3), *n* (%)	38 (35.5)	24 (45.3)	10 (30.3)	4 (19)	0.114
Favorable Outcome (0–2 mRS score 3 months), *n* (%)	34 (31.8)	16 (30.2)	12 (36.4)	6 (28.6)	0.579

There were no other significant differences across the groups regarding primary angiographic or clinical outcomes. Our subgroup analysis of SR treatment highlighted higher FPE in the 6 × 50 SR (37.7%), than in the rest of the SR which are 21.2% (4–5 mm size and 20–50 mm length SR) and 19% (6 mm size and 25–40 mm length SR), but it was not found to be statistically significant. There was no statistically significant association between technical approaches and procedural safety variables.

## Discussion

This study included 121 patients with acute intracranial ICA occlusion treated with either an SR or DA. The rate of successful recanalization of the ICA occlusion after the first pass was 25.6%. A previous systematic review and meta-analysis demonstrated similar success rates, with a mean of 26.2% (10). We found a clear superiority in obtaining an FPE using SR compared to DA in out cohort. Additionally, the data suggest that higher success rates are associated with larger stent retrievers.

Only three studies have compared SR and DA in distal ICA occlusions; two of them demonstrated the superiority of SR (11, 12), whereas the third study revealed that DA is superior to SR if BGC was not used (13). Our data showed the superiority of SR over DA (FPE 29 vs. 0%; *p* = 0.020). One possible explanation for these results is that carotid terminus occlusions have larger thrombus burdens and distal aspiration catheters may not have the optimal size for the vessel diameter, which can be 3.6 ± 0.4 mm at the ICA terminus and 5 ± 0.6 mm at the cavernous segment (14). New large-bore aspiration catheters with a larger inner diameter (≥0.072 mm) (15) attempt to mitigate this issue. Another reason may be that the aspiration catheter must align perfectly with the thrombus, which may be difficult in tortuous anatomy.

In intracranial occlusions of the ICA, the thrombus may extend into the anterior cerebral artery or middle cerebral artery. If the total length of the thrombus is unknown, a longer device allows more segments to be covered with a lower chance of losing the target site. Additionally, this modification allows for a greater device/thrombus interaction, and the larger diameter maintains greater entrapment during clot traction (16). As a result, there is a greater chance of FPE and a reduction of embolisms to new territories.

Our subgroup analysis of SR treatment highlighted higher FPE and mFPE in the 6 × 50 SR (37.7 and 45.3%, respectively) than in the rest of the SR, but it was not found to be statistically significant. We believe that the failure to show significance was due to the small sample size. Previous comparisons between 4- and 6- mm diameter Solitaire stent retrievers also did not find differences in treatments for ICA occlusions, but they did not evaluate FPE and only focused on reperfusion TICI ≥ 2b or TICI ≥ 2c (17). Similarly, in the Systematic Evaluation of Patients Treated With Neurothrombectomy Devices for Acute Ischemic Stroke (STRATIS) Registry, the SR diameter did not significantly affect the results of ICA recanalization (18).

Our results highlight a significant difference in the mFPE as fixed 6 mm diameter stents with the longest length were the most effective (45.3% in >50 mm and 19% in <50 mm; *p* = 0.037). These results are in accordance with previous literature, where length was the most important parameter in both *in vitro* studies of fibrin-rich clots (19), *in vivo* studies, and registries (18, 20).

In our multicenter study, a BGC was only used in 37.2% of cases. A recent systematic review showed benefits in the use of BGC with DA and SR but not with the combined technique (21), although data from the ASTER2 trial results suggest better efficacy in distal ICA occlusion when the combined approach is performed with BGC (22).

Our study has some limitations, as procedural outcomes were reported by neurointerventionalists without an independent core lab, which can lead to a reporter bias. There is also a lack of statistical power regarding the type of ICA occlusion that correlates with different collateral flow patterns and subsequent clinical outcomes (23, 24). We have not reported other etiologies either, except tandem occlusion, since it has been difficult to define whether it is reliable given the retrospective nature of the study. This study has other limitations, including the fact that it was a retrospective analysis and a multicentric study involving different procedural methodologies that might have influenced the results. For example, the low use of BGC might have an influence on the low rate of FPE. However, having different neuroendovascular approaches more realistically simulates the preferences of different neurointerventionalists, mirroring the real procedural practice. Likewise, it is an important limitation that the sample is unbalanced between stentriever and direct aspiration groups.

Since the most effective endovascular treatment is still unknown, future studies should include a larger number of patients as well as other possible technical approaches to recanalize an ICA occlusion, such as the use of the double-stent retriever technique or the association of BGC, larger-bore distal aspiration catheters, and the stent retriever.

## Conclusion

In our intracranial ICA occlusion series, stent retrievers were superior to direct aspiration in obtaining FPEs and mFPEs, and longer devices achieved better results. A trend toward FPE was observed when using the 6 × 50 stent retriever. Further studies evaluating the effects of different ICA clot removal approaches are warranted to confirm these results.

## Data Availability Statement

The raw data supporting the conclusions of this article will be made available by the authors, without undue reservation.

## Ethics Statement

The studies involving human participants were reviewed and approved by Comité de Ética de la Investigación con medicamentos del Hospital Clinic de Barcelona (HCB/2020/1422). Written informed consent for participation was not required for this study in accordance with the national legislation and the institutional requirements.

## Author Contributions

DH, ES, OC, MW, and AL-R: conceived and designed the analysis, collected the data, contributed data or analysis tools, performed the analysis, and wrote the paper. GM and FZ: conceived and designed the analysis, contributed data or analysis tools, performed the analysis, and critical review of the manuscript with intellectually relevant contributions. BL, AR, LL, MC, SR, and CP: collected the data, contributed data or analysis tools, performed the analysis, and critical review of the manuscript with intellectually relevant contributions. MR: collected the data, contributed data or analysis tools, performed the analysis, and wrote the paper. All authors have reviewed and approved the manuscript.

## Conflict of Interest

The authors declare that the research was conducted in the absence of any commercial or financial relationships that could be construed as a potential conflict of interest.

## Publisher's Note

All claims expressed in this article are solely those of the authors and do not necessarily represent those of their affiliated organizations, or those of the publisher, the editors and the reviewers. Any product that may be evaluated in this article, or claim that may be made by its manufacturer, is not guaranteed or endorsed by the publisher.
